# Maximizing Participation in Olfactory Training in a Sample with Post-COVID-19 Olfactory Loss

**DOI:** 10.3390/brainsci14070730

**Published:** 2024-07-21

**Authors:** Alice Helena Delgado-Lima, Jaime Bouhaben, María Luisa Delgado-Losada

**Affiliations:** Experimental Psychology, Cognitive Processes and Speech Therapy Department, Faculty of Psychology, Complutense University of Madrid, 28223 Pozuelo de Alarcón, Spain; alicedel@ucm.es (A.H.D.-L.); jaimebou@ucm.es (J.B.)

**Keywords:** olfactory training, olfactory dysfunction, therapeutic approach, olfactory rehabilitation, COVID-19

## Abstract

Purpose: This study aims to highlight the feasibility of an olfactory training program entirely monitored through online media in COVID-19 patients. Methods: Classic olfactory training was performed with a sample with olfactory loss due COVID-19 (*n* = 11). Participants were engaged on a weekly video call in order to improve adherence and collect information regarding the number of correct answers and the individuals’ perception of olfactory function. The olfactory status after training was compared to two groups, one composed of participants who contracted COVID-19 but did not report olfactory loss (*n* = 11) and a sample composed of healthy participants (*n* = 11). Results: The experimental group showed improvements throughout the training period (TDI score on week 0 was 20.3 (5.6) and 24.6 (4.3) for week 12, and on week 24 was 25.4 (6.2) (F = 5.115, df = 2, 20, *p* = 0.016), and post hoc tests showed that participants significantly improved their TDI score in W12 compared to W0 (SMD = 0.869, *p* = 0.041) and in W24 compared to W0 (SMD = 0.859, *p* = 0.041). The experimental group showed lower scores when compared with both groups, and the no OT COVID-19 group showed lower scores than the healthy control group, even though they did not report olfactory alterations. Conclusions: Findings suggest that the strategies applied to improve adherence were successful since 100% of the sample completed the training adherence, offering a valuable framework for future olfactory training studies.

## 1. Introduction

The coronavirus disease 2019 (COVID-19) is an infection caused by severe acute respiratory syndrome coronavirus 2 (SARS-CoV-2) and is so far responsible for more than 6 million deaths and more than 700 million people worldwide having been infected [1]. The most common symptoms that may suggest COVID-19 include fever, fatigue, cough, dyspnea, myalgia, arthralgia, diarrhea, and headache. Bilateral pneumonia is frequently seen on chest radiographs or computed tomography scans, along with bilateral pulmonary involvement. Sudden olfactory loss, anosmia (complete loss of smell), or hyposmia (partial loss of smell) have been reported as symptoms of COVID-19, presenting as an initial symptom, concomitantly or immediately after general symptoms [2,3,4]. Some degree of olfactory dysfunction (OD) is estimated to be present in a larger majority, with up to 85–98% of patients affected in some studies [5,6,7,8]. The natural course of OD due to COVID-19 is spontaneous resolution by two weeks for 95% of patients, with mean recovery of 9 days [8], with most patients regaining their sense of smell within 14 days of resolution of COVID-19 infection (72.6% within 8 days), and only 3.3% of the cases with hyposmia, and 3.4% with anosmia, took more than 15 days [3,9,10,11,12]. As reflected by the percentages observed, a large proportion of people recover their sense of smell within weeks. However, up to 5% report persistent olfactory problems beyond the initial weeks of COVID-19 infection, including anosmia, hyposmia, parosmia, and phantosmia [7,13]. Some studies indicate that patients may continue to experience symptoms of olfactory dysfunction for one to two years or even longer following a COVID-19 infection [14,15,16,17,18,19] and OD was found to be a persistent and a prevalent symptom, appearing in 75% of cases with persistent COVID symptoms [14]. Research has shown that SARS-CoV-2 can impair olfactory function in patients with long COVID by triggering a chronic inflammatory response, which both damages olfactory sensory neurons and hinders their regeneration [20].

SARS-CoV-2 uses the binding of its spike proteins with a receptor Angiotensin-converting enzyme (ACE2) and the proteolytic processing of spike proteins for cell entry [21]. ACE2 is the functional receptor for SARS-CoV-2 [22]. Considering that the respiratory epithelium is the primary site of infection for SARS-CoV-2 and many other viruses, it is unsurprising that COVID-19 also impacts the olfactory neuroepithelium [23]. After attaching to the respiratory tract, SARS-CoV-2 travels down the airways and infiltrates alveolar epithelial cells in the lungs, triggering an inflammatory response [21]. Different investigations have been made to understand the specific cell types within the olfactory epithelium and the brain that are affected by SARS-CoV-2. These studies show that ACE2 expression, both at the gene and protein levels, is limited to non-neuronal cell types such as sustentacular cells, mucus-secreting Bowman’s gland cells, stem cells like horizontal basal cells, and vascular cells [24,25]. SARS-CoV-2 causes a cytokine storm that can cause damage to the nervous system, including olfactory receptors [26].

Research involving both model organisms and autopsies from COVID-19 patients has convincingly demonstrated that sustentacular cells are the primary target of SARS-CoV-2 in the olfactory neuroepithelium. This suggests that anosmia in COVID-19 patients is not caused by neuronal infection or neuroinvasion [26,27].

While SARS-CoV-2 may affect both the olfactory and gustatory systems, in most non-COVID-19 cases where patients report altered taste, this symptom is often due to impaired retronasal olfaction (flavor) rather than true gustatory impairment (sweet, salty, sour, bitter) [28].

OD is known to have a significant impact on quality of life, since it diminishes a person’s ability to enjoy foods and fragrances [19,29,30]. The sense of smell helps recall olfaction-associated memories and detect hazardous materials such as spoiled food and toxic fumes. Furthermore, olfactory dysfunction is linked to various debilitating psychosocial effects, including depression, social isolation, impaired cognition, decreased nutrition, and increased mortality [31].

The management and mitigation of OD caused by COVID-19 infection have become crucial in improving the overall quality of life for affected individuals [32]. Given the significant impact of this sensory impairment, various pharmacological treatment options have been proposed with the aim of addressing and ameliorating OD symptoms, such as intranasal corticosteroid sprays [33,34]. In addition to pharmacological treatments, another approach that has gained attention for mitigating the effects of COVID-19 on the olfactory system is olfactory training (OT). This non-pharmacological therapeutic approach is based on the regeneration capacity of olfactory receptor neurons and/or improved higher order processing of olfactory information [35,36]. 

OT is a novel, non-invasive approach aimed at rehabilitating olfactory dysfunction. Accumulating evidence indicates its efficacy in treating individuals experiencing impaired sense of smell caused by diverse factors [31,36,37,38,39,40].

This therapeutic intervention involves targeted exercises designed to stimulate and rehabilitate the olfactory system; by engaging in repetitive exposure to specific odors, individuals aim to regain their sense of smell and improve olfactory function. The method consists of people sniffing four different odors, usually one odor of the categories: flowery, fruity, spicy, and resinous. Such training is performed twice a day for a certain time period (usually between 4 and 6 months). Hummel et al. pointed out that a structured, short-term exposure to odorants over 12 weeks increased olfactory sensitivity in 28% of participants [41], OT was also found to be an effective approach in different studies [32,37,39]. Despite its potential benefits, maintaining participant adherence in OT programs has been a significant challenge, as reported in different studies [42,43,44,45,46]. Dropout rates and inconsistent participation can undermine the effectiveness of OT, leading to less reliable outcomes and limiting the generalizability of findings.

This study aims to conduct a follow-up assessment with the experimental group undergoing olfactory training as well as to test the feasibility of an olfactory training entirely monitored through online media.

## 2. Materials and Methods

### 2.1. Participants

Participants were from Complutense University of Madrid and Madrid Association of Persistent COVID. Olfactory evaluations and olfactory training were conducted between June 2021 and June 2022.

To facilitate the investigation, were divided into three distinct samples(s):

S1 training group: referred to as the experimental group (EG), consisted of 11 participants (mean age 34.55 ± 11.88; 6 women and 5 men). These participants reported OD and were actively involved in the OT intervention.

S2 COVID-19 untrained group: designated as no OT COVID group (CG), included 11 participants (mean age 33 ± 11.43; 6 women and 5 men). These participants had previously contracted COVID-19 but did not report any olfactory symptomatology nor OD. They did not partake in the OT intervention.

S3 healthy untrained group: referred to as healthy group (HG), comprised 11 participants (mean age 32.55 ± 11.74; 8 women and 3 men). These participants had not contracted COVID-19 and did not experience OD. They were not involved in the OT intervention.

The inclusion criteria for sample 1 or experimental group were as follows: (i) to be 18 years or older, (ii) absence of current otorhinolaryngology alterations, previous COVID-19 diagnosis (self-reported), (iii) persistence of OD at 3 months post-COVID-19, (iv) confirmation of OD with a TDI score assessed with Sniffin’ Sticks < 22 (this score corresponds to the 10th percentile in the Spanish population [32], a percentile used to discriminate between hyposmia and normosmia [33], (v) time elapsed from diagnosis to intervention less than 12 weeks, and (vi) compliance with testing procedure. While for sample 2 and 3 the inclusion criteria were: (i) to be 18 years or older, (ii) absence of current otorhinolaryngology and olfactory alterations, and (iii) compliance with testing procedure. 

However, the exclusion criteria were for all samples: (i) a medical history of olfactory alterations, including nasal polyposis, sinusitis, congenital olfactory loss, prior history of olfactory loss or prior nasal surgery, (ii) medication intake with repercussion in olfactory performance (such as some antibiotics, antiepileptics, antithyroids, benzodiazepines, or antiarrhythmics, antidiabetic medication), (iii) clinical history of ENT radiotherapy (iv) presence or suspicion of cognitive impairment and/or neurologic or psychiatric dysfunctions, and (v) self-declared pregnancy [47].

### 2.2. Measures and Procedures

The assessment protocol was composed of a sociodemographic questionnaire and an olfactory evaluation.

Sociodemographic questionnaire: A questionnaire survey was fulfilled by participants in order to collect sociodemographic and clinical information related to health, smoking habits, and prior olfactory status and COVID-19 previous diagnoses. Due to the health situation when data were collected, information about COVID-19 previous diagnoses was also obtained and analyzed.

Olfactory performance evaluation: The complete version of the Spanish adaptation of the Sniffin’ Sticks Olfactory Test (SSOT) (Burghart Messtechnik GmbH, Wedel, Germany) was adapted to the Spanish population by Delgado-Losada et al. [32]. The complete version includes three tests that aim to measure different components of olfactory function, namely, including olfactory threshold (T), odor discrimination (D), and odor identification (I). Each test gives a unique score, ranging from 0 to 16, representing each olfactory component, and it may also be administered independently. The sum of the three odor scores (T, D, and I) defines a composite score (TDI, ranging from 0 to 48), which measures general olfactory function. Olfactory function was assessed for both nostrils. For odor presentation, pens with a length of 14 cm and a diameter of 1.3 cm were used. Each pen was filled with 4 mL of the corresponding liquid odorant. The evaluator took the pen’s cap off and put the tip of the pen in front of the participant’s nostrils, with an approximate distance of 2 cm. In any case, the tip of the pen never physically touched the participant’s nose. In all the three tests, each odor pen was presented to the participant for 3 s. The overall time of administration ranged from 30 to 45 min, depending on how long the T subtest lasted. The order of test presentation is T, D, I, as in the original version. The administration procedure follows the one established in this original version [33]. In addition, the original test set the 10th percentile as the cutoff point for hyposmia [33]. These criteria are also taken into account in this present study. The Spanish validation reports the 10th percentile in different age cohorts for each score as the cutoff point for hyposmia, as well as for TDI [32].

Participants underwent testing in a tranquil, well-ventilated environment to prevent any ambient odors from affecting the test odors, and they wore odorless gloves. All participants were instructed to refrain from consuming food, beverages, smoking, chewing gum, applying cologne, or brushing their teeth for up to one hour prior to the test (though drinking water was permitted).

### 2.3. Experiment Design

#### 2.3.1. Experiment 1: Olfactory Training

To conduct the first experiment for this investigation, the experimental group was submitted to an olfactory evaluation to draw their baseline for olfactory capacity prior to OT. In this first psychophysical evaluation, the participants were given an olfactory training kit containing the 4 traditional odors proposed by Hummel et al. [31] (rose, eucalyptus, lemon, and cloves). OT in this study was self-administered by participants. Prior to commencing the training, participants were provided with detailed information regarding the specific smells included in the training kit ensuring that participants were familiar with the range of odors they would encounter. Participants were asked to expose themselves to each odorant for at least 10 s, rotating through all 4 odors to finish the set. During the training sessions, participants conducted the olfactory exercises and subsequently verified their responses by checking the labels at the bottom of each odorant container. With each set, they had to name each odorant and report the number of correct answers for each training session on an Excel spreadsheet shared with a member of the investigation group. For every training session, the participant could obtain a maximum of 4 correct answers, which would lead to 8 correct answers daily and a maximum of 56 correct answers per week.

To maintain the integrity and consistency of the olfactory stimuli, the odor kits were procured from a certified supplier and each kit included a specified expiration date. This precaution ensured that the quality of the odors remained consistent throughout the study period. Additionally, participants were provided with explicit storage instructions, aligned with the manufacturer’s recommendations, to prevent any alteration or degradation of the smells.

Throughout the study, participants were scheduled for weekly virtual calls with a team member. These calls served to collect relevant information pertaining to their OT progress. During these sessions, participants were asked to report the number of correct answers achieved in their OT. Additionally, participants were requested to provide a subjective evaluation of their olfactory capacity using an analog scale ranging from 0 (complete absence of the sense of smell) to 10 (no olfactory alterations). Moreover, participants used a shared spreadsheet with a member of the research team to record their scores from each training session. This method was applied in order to collect participants’ views on olfactory capacity regarding his daily life and the challenges OD presents, collect personal information regarding olfactory changes observed by the participant and also to improve adherence to the OT, since this was a challenge reported by different investigations [31,34].

The OT was performed for a total of 24 weeks. 

The experimental group was evaluated on week 0 (initial evaluation and prior to OT), week 12 (follow-up evaluation), and week 24 (final evaluation).

#### 2.3.2. Experiment 2: Comparative Analysis

In order to comprehensively examine the influence of COVID-19 on the olfactory system, the analysis of results obtained from the experimental group after olfactory training was conducted in comparison with two additional groups.

The first group consisted of individuals who had contracted COVID-19 but did not report or observe any olfactory alterations. The second group comprised individuals who had not contracted COVID-19. By comparing the experimental group’s outcomes to this control group, the study aimed to discern the unique impact of COVID-19 on olfactory function, separate from any potential factors unrelated to the infection.

Additionally, comparisons were drawn between all 3 samples aiming to better understand the influence of COVID-19 on the olfactory system.

### 2.4. Study Design

The design for this investigation is a quasi-experimental design without random allocation. The experimental group underwent an intervention involving olfactory training, while the other groups did not receive this intervention. To analyze the effects of OT on olfactory capacity, the experimental group was evaluated at three different time points. The assessment procedure spanned multiple time points to capture changes in olfactory capacity resulting from the OT intervention.

During the first visit, all participants completed a sociodemographic questionnaire, and the Sniffin’ Sticks Olfactory Test was administered. 

This study was ruled by the principles of the Declaration of Helsinki (Edinburgh, 2013) and was approved by the Ethics Committee from University Hospital San Carlos (Madrid, Spain) (ref. number 20/515-E). The study was adjusted to standards of good clinical practice (art.34 RD 223/2004; community directive 2001/20/CE), and to the protection of personal data and confidentiality (European Data Protection Regulation, and in accordance with the Organic Law 3/2018 on the Protection of Personal Data and Guarantee of Digital Rights).

### 2.5. Statistical Analysis

The statistical analysis in this study utilized Python 3.8.10 software (Python Software Foundation, Wilmington, DE, USA, 2021) for data processing and computations. The results are presented as means ± standard deviation (SD).

In the first experiment, descriptive analysis was conducted on the sample undergoing OT. Mean and standard deviation were calculated for age and olfactory status variables (T, D, I, and TDI) and Shapiro–Wilk test was performed to check normality. To assess changes in olfactory function across evaluation periods (W0, W12, and W24), analysis of variance (repeated measures design: rm-ANOVA) was performed. Greenhouse–Geisser correction due to lack of sphericity was applied. The number of weekly correct answers during the OT period and participants’ weekly subjective perception of olfactory function in daily life were also analyzed, with means and standard deviations calculated.

In the second experiment, comparisons were made between all samples to determine the impact of OT and the status of EG after OT. Analysis of variance (one-way ANOVA) was employed for these comparisons. Welch’s correction was applied due to the risk of unequal variances. Post hoc between-group multiple comparisons were conducted using Tukey’s honestly significant difference (HSD) test to identify any significant differences.

## 3. Results

### 3.1. Experiment 1: Olfactory Training

Experiment 1 focused on sample 1, which represents the experimental group undergoing OT. The objective of this experiment was to analyze the olfactory changes experienced by the group following the OT intervention. To capture the dynamics of these changes, the experiment consisted of three measuring periods: week 0, week 12 (follow-up), and week 24 (after the completion of OT). Week 0 served as the baseline measurement, providing insight into the participants’ initial olfactory capacity prior to any intervention. While week 12 served as a follow-up assessment to evaluate the progress and observe changes in olfactory function after 12 weeks of OT. Lastly, week 24 was the final measuring period, allowing for the assessment of long-term effects and sustained improvements in olfactory capacity following the completion of the OT program. By examining olfactory changes over these three time points, this experiment aimed to provide a comprehensive understanding of the impact of OT on the olfactory function of sample 1.

Descriptive analysis of the experimental group by olfactory subtests performance is shown on Table 1. Sample 1 was composed of 11 participants with a mean age of the group 34.55 (11.88), (six women with an average age of 38.67 ± 11.27 and men with an average age of 28 (11.27)). There was no evidence of non-normality for any olfactory variable (TDI: W = 0.963, *p* = 0.316; T: W = 0.961, *p* = 0.288; D: W = 0.947, *p* = 0.111; I: W = 0.938, *p* = 0.061).

For olfactory threshold (T) on week 0, the mean score was 3.8 ± 3.3 and 6.2 (3.8) for week 12 and on the final evaluation (week 24) 6.5 (3.4). Single factor ANOVA model with repeated measures (rm-ANOVA) was performed to assess potential changes between assessment moments. No statistically significant differences were found in T olfactory threshold, however (F = 3.368, df = 2, 20, *p* = 0.055).

For olfactory discrimination (D) on week 0, the mean score was 7.5 (3.4) and 8.9 (3.2) for week 12 and on the final evaluation (week 24) 8.3 (3.1). Single-factor ANOVA model with repeated measures (rm-ANOVA) was performed, but no statistically significant differences were found between time moments regarding D (F = 0.647, df = 2, 20, *p* = 0.534).

For olfactory identification (I) on week 0, the mean score was 8.9 (1.3) and 9.5 (2.1) for week 12 and on the final evaluation (week 24) 10.5 (2.3). Another single factor ANOVA model with repeated measures (rm-ANOVA) was calculated, but no significant differences were found between time moments (F = 2.19, df = 2,20, *p* = 0.138).

Finally, for TDI score on week 0, the mean score was 20.3 (5.6) and 24.6 (4.3) for week 12 and on the final evaluation (week 24) 25.4 (6.2). Single-factor ANOVA model with repeated measures (rm-ANOVA) yielded a statistically significant difference (F = 5.115, df = 2, 20, *p* = 0.016) between temporal measures. Post hoc, *p*-value corrected, and multiple comparison tests were then performed: patients significantly improved their TDI score in W12 compared to W0 (SMD = 0.869, *p* = 0.041) and in W24 compared to W0 (SMD = 0.859, *p* = 0.041). No statistically significant improvement was found between W12 and W24 in TDI score (SMD = 0.136, *p* = 0.657).

**Figure 1:**
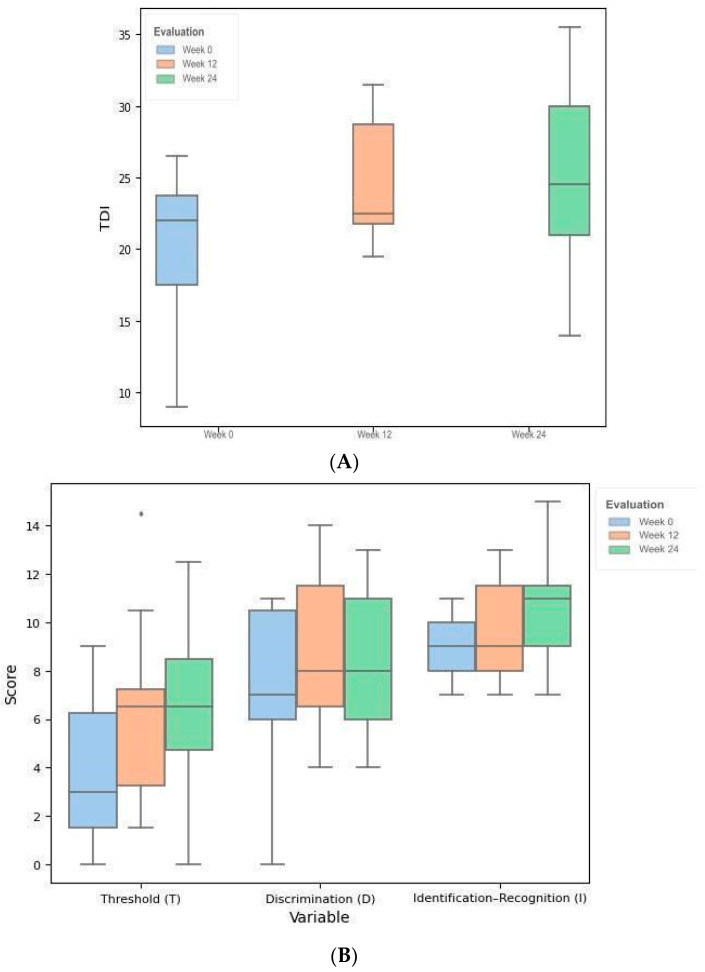

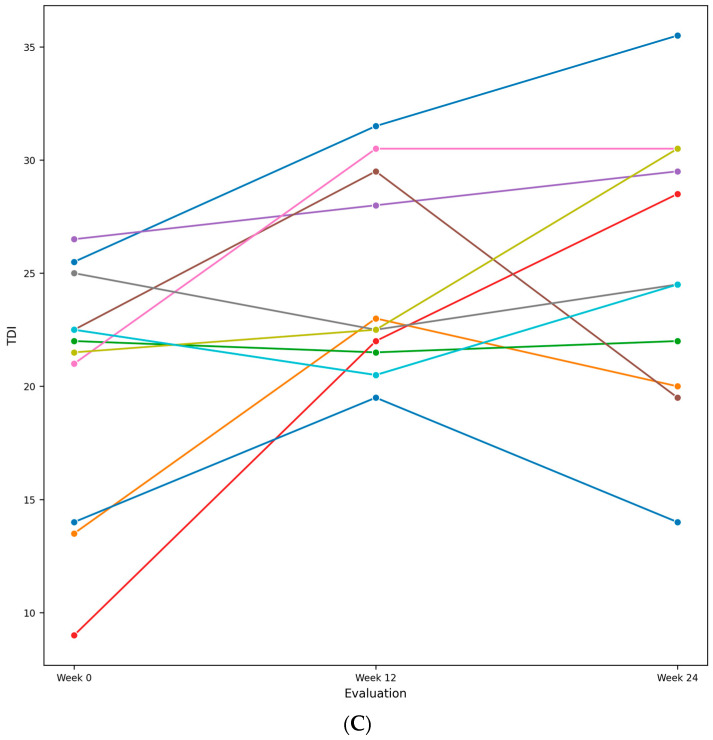
(**A**). Boxplot of TDI comparison between evaluation periods of the EG. (**B**). Boxplot of olfactory subtests comparison between evaluation periods of the EG. (**C**). Line plot of participants’ evolution from the EG.

In Figure 1A,B, the progress of the olfactory performance throughout the OT is shown, while Figure 1C presents the evolution of each participant through the OT period.

**Figure 2:**
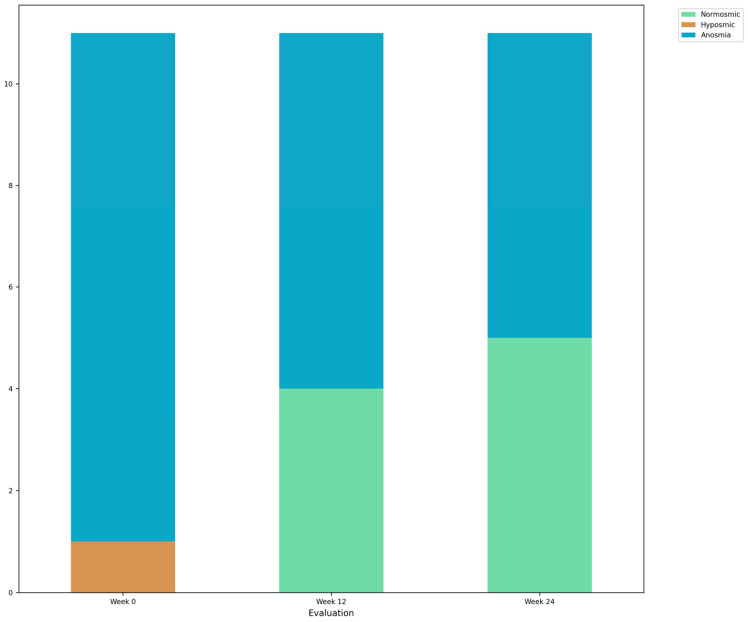
Bar chart of the evolution of OD of the EG.

During the initial evaluation in week 0 (prior to OT), the entire sample exhibited OD (one hyposmic and ten anosmic). To classify the severity of OD within the sample, a classification system based on the Spanish validation reports for different age cohorts was utilized. This system employed the 10th percentile as the cutoff point for hyposmia, considering the respective scores for each age cohort [32]. By utilizing this classification system, the severity of olfactory dysfunction within the sample could be determined, providing a standardized framework for assessing the baseline levels of OD prior to the implementation of OT. The evolution of OD can be seen in Figure 2.

During week 12, the second evaluation was conducted to assess the short-term effects of OT. In this evaluation, out of the participants, seven of the participants classified as anosmic, whereas four progressed to normosmia. At the final evaluation in week 24, five participants were classified as normosmic, indicating a sustained improvement in olfactory function following the completion of OT. However, six remained anosmics.

In this experiment, participants engaged in a weekly video call with a member of the investigation team to provide information regarding their OT progress. Specifically, participants were asked to report the number of correct answers they achieved on a weekly basis. Additionally, they were requested to subjectively rate their olfactory perception within daily life activities on a scale from 1 to 10. Additionally, there was a shared spreadsheet between participants and a member of the investigation group, where participants recorded their daily OT results.

**Figure 3:**
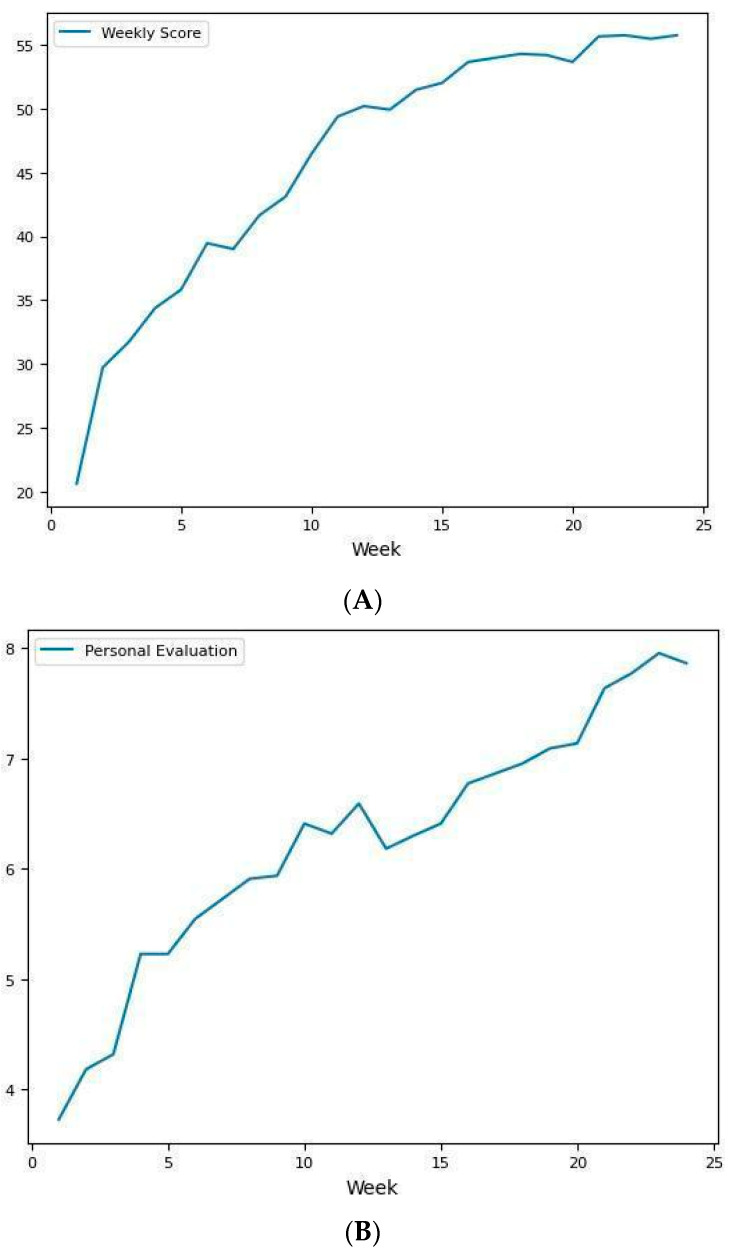
(**A**). Line chart showing the mean evolution of the number of correct answers through olfactory training. (**B**). Line chart of the evolution of the participants in the EG self-rate olfactory performance per week.

Figure 3A illustrates the evolution of the mean number of correct answers per week of training during the OT period (SD = 11.733). This score is obtained from the online monitoring, which participants received during training. The data presented in this figure demonstrates a progressive increase in the number of correct answers during training time, indicating how participants are progressing in the intervention. This upward trend suggests a positive response to the OT intervention.

Figure 3B showcases the participants’ self-rated olfactory performance for each week (SD = 1.914). This score also is obtained from the online monitoring, which participants received during training. The data presented in this figure also exhibits an upward trend, indicating a subjective improvement in olfactory perception within daily life activities as reported by the participants.

### 3.2. Experiment 2: Comparative Analysis

Experiment 2 aimed to compare the olfactory performance of the experimental group (EG) with two groups: no OT COVID-19 group (CG), consisting of participants who contracted COVID-19 infection but did not report olfactory alterations (*n* = 11, six women, mean age = 33 ± 11.43), and healthy group (HG), composed of participants who did not contract COVID-19 (*n* = 11, eight women, mean age = 32.55 ± 11.74). There was no evidence of non-normality for any olfactory variable (TDI: W = 0.944, *p* = 0.091; T: W = 0.946, *p* = 0.108; D: W = 0.945, *p* = 0.098; I: W = 0.942, *p* = 0.077). Table 2 provides a descriptive analysis, presenting relevant information about the three groups regarding the participants’ characteristics and olfactory status.

**Figure 4:**
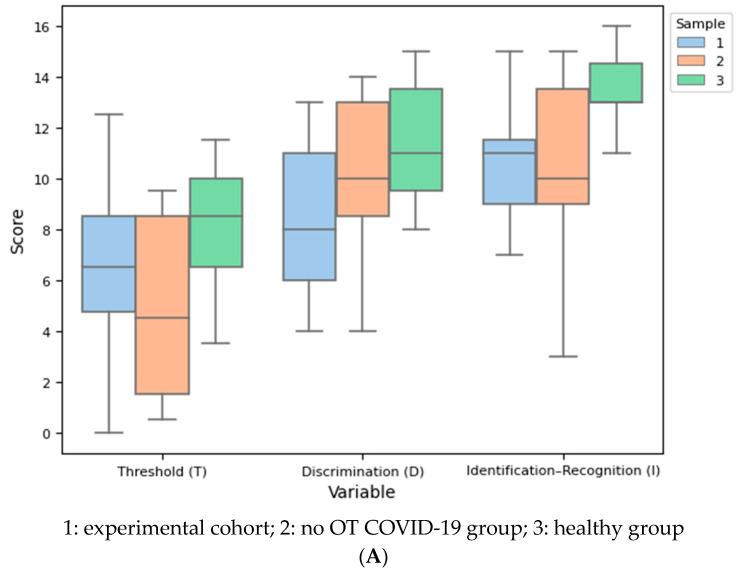

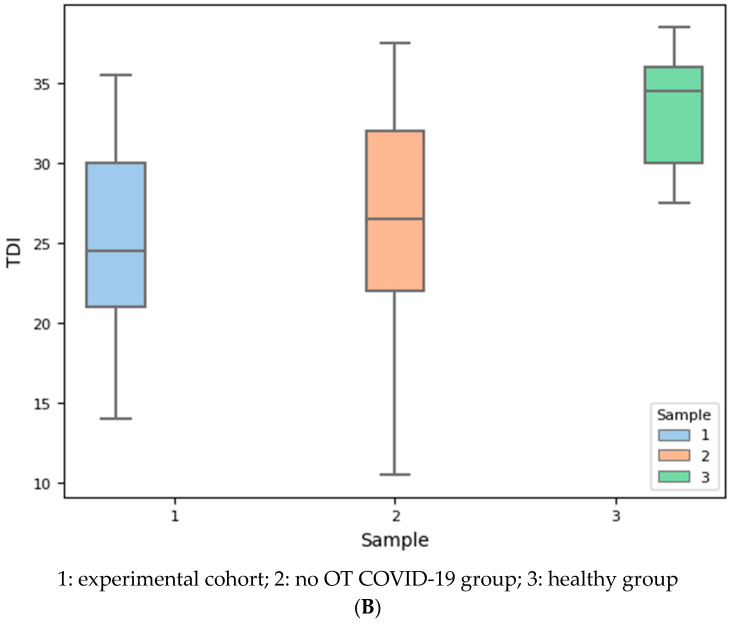
(**A**). Boxplot of comparison between olfactory subtests by sample. (**B**). Boxplot of comparison between TDI by sample.

Comparisons between COVID-19 status groups were calculated for each olfactory measure through one-way ANOVAs. Statistically significant differences were found in all tests favoring healthy group controls, who performed better in all olfactory subtests (T = 8.2 (2.5); D = 11.5 (2.3); I = 13.5 (1.4); TDI = 33.2 (4.0)). Although no OT COVID-19 control group did not report olfactory alterations, the group presented lower results when compared to the healthy group controls (T = 4.9 (3.5); D = 10.5 (3.3); I = 10.6 (3.5); TDI = 26.0 (8.6)), followed by the experimental group with lower scores (T = 6.5 (3.4); D = 8.3 (3.1); I = 10.5 (2.3); TDI = 25.4 (6.2). For each olfactory score, post hoc, p-corrected, and multiple comparisons were performed in addition to Tukey’s HSD tests. Figure 4A,B present the comparison between the samples by olfactory subtests and TDI score.

Firstly, regarding the T olfactory threshold (T, F = 2.928, df = 8, *p* = 0.069), no differences were found between HG and EG (SMD = 1.493, *p* = 0.055) nor HG and CG (SMD = 1.064, *p* = 0.456), nor CG and EG (SMD = −0.475, *p* = 0.456). Next, in D olfactory discrimination (D, F = 3.391, df = 8, *p* = 0.047), significant differences were found between HG and EG (SMD = 1.158, *p* = 0.042), but not between HG and CG (SMD = 0.352, *p* = 0.706) nor CG and EG (SMD = 0.685, *p* = 0.205). Furthermore, I olfactory identification (I, F = 4.903, df = 8, *p* = 0.014) comparisons show differences between HG and EG (SMD = 1.545, *p* = 0.026) and between HG and CG (SMD = 1.095, *p* = 0.032), both favoring the healthy group. The comparison of EG and CG is remarkable, as no differences in I olfactory identification were found (SMD = 0.031, *p* = 0.996) between COVID-19 patients with olfactory dysfunction and COVID-19 who did not report any olfactory symptom or issue. Finally, tests for TDI score, which summarizes olfactory function, were performed (F = 4.819, df = 8, *p* = 0.015). HG scored The comparison between EG and CG also stands out, as no statistically significant differences were found regarding olfactory performance between these two samples (SMD = 0.085, *p* = 0.972). 

Following the experiment, individuals in the no OT COVID-19 group, who were initially unaware of their post-COVID-19 olfactory impairment, were subsequently provided with olfactory training to address any potential ethical concerns regarding the omission of treatment.

## 4. Discussion

This study had two parts: first, to assess the sense of smell in a sample of individuals who experienced OD due to SARS-CoV-2 infection; and second, to evaluate their progress over a 24-week OT intervention. The first group comprised participants who contracted COVID-19 but did not perceive any olfactory alterations (no OT COVID-19 group). The second group consisted of healthy group who did not contract COVID-19.

Through this comparison, the study aimed to understand the impact of the OT intervention in improving olfactory function in the sample affected by COVID-19-induced OD. Additionally, by comparing the sample with the two groups, the study sought to differentiate the effects of COVID-19 on olfactory function from those attributed to the OT intervention or other factors unrelated to the infection.

Building on these objectives, this study incorporated innovative strategies to ensure high participant adherence and comprehensive data collection.

Our study ensured that all participants completed the olfactory training protocol. This high level of adherence can be attributed to our innovative strategy of incorporating weekly video calls with the participants. These regular video interactions provided continuous support, guidance, and motivation, significantly enhancing compliance with the training regimen. Additionally, we implemented a shared spreadsheet between each participant and a member of the investigation team, where participants recorded their daily olfactory training results. This dual approach allowed for real-time troubleshooting, making participants more likely to follow through with the training. This approach, in contrast with other studies where lack of adherence to olfactory training protocols is a common challenge [48,49,50], often leading to incomplete data and less reliable outcomes. 

This study was divided into two experiments. 

Experiment 1 was based on the experimental group, and it was conducted to evaluate the pre- and post-effect in olfactory measures after OT. Throughout the 24 weeks that OT was performed, it was observed a positive and significant effect in TDI score from baseline to the final evaluation, although the participants improved in the overall olfactory subtest, and the results present a positive trend (see Table 1), those differences were not statistically significant. Similar results were found in the investigation of Kollndorfer and colleagues [51], where after completing OT for a period of 12 weeks, participants (*n* = 7) showed a statistically significant sensitivity to detect odors (threshold), but such differences did not extend to the other olfactory scores. An analogous investigation by Hosseni et al. (2020) [52] observed significant improvements in olfactory performance following a 16-week smell training regimen. Statistically significant differences were noted when comparing the TDI score and discrimination abilities of the experimental group before and after the training period. Another investigation with similar results found a significant threshold and identification improvement, followed by a non-significant discrimination improvement [53]. On the same lines, Yaylaci and colleagues (2022) found that the experimental group improved significantly in all olfactory subtests after 12 weeks of OT [54].

Contrasting the results found in Altundag et al. (2015), where participants on the conventional olfactory training group showed significant improvement in both discrimination and identification tasks but not on the threshold nor TDI score [55]. Nonetheless, previous work regarding OT has observed that the gain in odor thresholds is also involved in the improvement seen after OT [56]. 

In this investigation, participants showed a positive trend in all olfactory subtests, although like the investigation of Kollndorfer and colleagues [51] such differences were more pronounced in threshold. This phenomenon warrants careful consideration within the context. One potential explanation for the disparity in improvement across olfactory measures is the inherent sensitivity of olfactory threshold to change. Olfactory threshold, representing the ability to detect the lowest concentration of a scent, may inherently exhibit greater responsiveness to intervention or training compared to discrimination and identification abilities. This sensitivity could stem from the physiological mechanisms underlying scent detection, where subtle changes in sensory perception may be more readily discernible over time.

One of the main difficulties associated with OT is adherence to the treatment, and different approaches have been suggested in order to improve it [55,57]. To the best of our knowledge, our study is the first to incorporate a novel weekly follow-up video call and a shared spreadsheet to enhance adherence to the OT program. This innovative approach provided valuable insights into the progress of OT, enabling us to gather important data on the cumulative number of correct answers achieved by participants on a weekly basis; additionally, we obtained participants’ weekly subjective ratings of their olfactory capacity.

This proactive method of follow-up allowed us to visualize and track the evolving impact of OT on participants’ olfactory function (see Figure 3). By regularly assessing both objective and subjective measures, we gained a comprehensive understanding of the participants’ responses to the intervention over time. The number of correct answers fluctuated from a mean of 18.81 on week 1 and a mean of 55.72 on week 24. Participants presented weekly improvements both on the number of correct answers and their personal rating for the sense of smell. 

Usually, the recommended training period is 12 weeks [41]. Yet, a subsequent multi-center follow-up study [57] demonstrated even greater enhancement in olfactory performance following a training period lasting a minimum of 18 weeks. Thus, studies investigating the effects of longer training periods have followed [39]. In this study, participants engaged in a 24-week OT program. However, the results indicated that there were no statistically significant differences between the OT’s effects at W12 and W24. Based on these findings, it is suggested that OT programs should have a minimum duration of 12 weeks to observe an overall improvement in olfactory function. The lack of significant differences between W12 and W24 implies that the benefits of OT may stabilize after 12 weeks.

Previous studies have shown that olfactory training can lead to partial recovery of olfactory function in patients experiencing olfactory dysfunction [57]. This study observed consistent improvements in olfactory performance after each evaluation section, and notably, the TDI scores exhibited statistically significant differences following the training period. These findings lead us to posit that the remarkable functional plasticity of the olfactory system [58] plays a pivotal role in the success of olfactory training, as evidenced by the significant increase in the TDI score. 

Experiment 2 consisted of the comparison of the experimental group with a no OT COVID-19 group and a healthy group. The objective of this comparison was to gain insights into the impact that COVID-19 has on the sense of smell.

Despite the improvement observed in experiment 1 among the participants in the experimental group, they still lag in terms of olfactory status compared to both groups. This discrepancy is evident in the lower scores obtained by the experimental group across all olfactory subtests (refer to Table 2), since statistically significant differences were only found regarding identification subtest and TDI score.

Similar findings were reported in the investigation conducted by Oleszkiewcz and colleagues [59], where an improvement was observed in the experimental group. However, despite the smell perception enhancement, statistically significant differences persisted when compared to the healthy group. Leading to the understanding that while improvements were observed in the experimental group, there is still a considerable gap between their olfactory status and that of the other groups. This discrepancy can be better understood by examining the mechanisms of SARS-CoV-2 infection and its impact on olfactory function. SARS-CoV-2 affects olfactory function by targeting cells in the olfactory epithelium, such as sustentacular cells and horizontal basal cells, which express the ACE2 receptor and TMPRSS2 necessary for viral entry [60]. This interaction causes inflammation and cell damage, disrupting the support cells crucial for the function of olfactory sensory neurons without directly infecting them [61]. Consequently, COVID-19 patients may experience olfactory dysfunction [24]. These disruptions can lead to persistent olfactory issues, even after the acute infection phase [26]. 

To further support these results, another study conducted by Kollndorfer et al. (2015) [51] investigated OT in a group of participants diagnosed with OD caused by upper respiratory tract infection. Despite the participants showing improvement after the training period, they still exhibited significant differences when compared to the healthy group. 

To the best of our knowledge, this study represents the pioneering investigation focused on olfactory loss after COVID-19 infection, wherein a no OT COVID-19 group with no complaints of olfactory loss has been included. By incorporating this no OT COVID-19 group, our research aims to shed light on the specific impact of COVID-19 on olfactory function, allowing us to draw more robust conclusions regarding the relationship between COVID-19 infection and olfactory loss. 

Despite the better results of no OT COVID-19 group obtained when comparing with the experimental group, they performed worse than the healthy group, in spite of not referencing olfactory disturbances after contracting COVID-19. This phenomenon can be attributed to the entry path of the SARS-CoV-2 virus. As the primary site of SARS-CoV-2 attachment and infection is the respiratory epithelium, it is plausible for COVID-19 to affect the olfactory neuroepithelium; and consequently, this impact on the olfactory system can lead to alterations in smell and flavor perception [62]. Disruption of cells within the olfactory neuroepithelium can lead to inflammatory alterations that hinder the function of olfactory receptor neurons, potentially causing damage to these neurons and impairing neurogenesis. These changes could result in temporary or prolonged olfactory dysfunction [28]. There is speculation that SARS-CoV-2 might invade the brain intracranially, affecting both olfactory and non-olfactory regions, which could negatively impact olfactory function. Additionally, not all COVID-19 patients were subjected to an objective evaluation of the olfactory capacity, leaving a considerable number of dysfunctions to go undiagnosed [28] and taking into consideration that psychophysical assessment involves presentation of odorants, with test outcomes dependent on the patient’s response being more reliable than a subjective assessment alone [28], supporting the idea that the prevalence of OD in COVID-19 is higher than it was originally speculated. Thus, psychophysical assessment enhances our understanding of the true extent of olfactory impairments related to COVID-19, contributing to a more accurate picture of the impact of the virus on the olfactory system.

While the study provides valuable insights into the therapeutic approach of OT, caution should be exercised when interpreting the findings due to the following limitations. In the first place, the lack of a randomized control group does not allow to establish a causal relationship between olfactory training and changes in olfactory function. Further study would require a sample of participants with olfactory dysfunction but who did not receive olfactory training. Allocation in experimental or control conditions should be properly randomized. Nonetheless, different studies have been developed without a control group [63,64,65,66,67]. Another limitation is sample size, as small sample sizes can lead to increased variability in the data, potentially influencing the results in unpredictable ways. However, olfactory training studies with limited sample sizes often appear in the similar literature [68,69,70]. Small sample size might also explain why no differences were found in T, D and I, although sample means showed increases from week 0 to week 12 and 24. T, D and I ranged from 0 to 16, thus this narrow range combined with limited sample size might mask statistical differences. Despite limitations, the present study might be an interesting starting point to administer an olfactory intervention whose compliance could be monitored through online media. 

## 5. Conclusions

Taken together, these findings suggest that OT may not have directly influenced individual olfactory subtests. However, the overall improvement in olfactory performance, as evidenced by the significant changes in the TDI scores, indicates that OT may have produced changes leading to an enhanced perception of odors.

This research obtained one hundred percent of adherence, suggesting that weekly video calls and shared spreadsheets are effective tools for maintaining participant engagement in long-term interventions. Notably, this research achieved 100% adherence, demonstrating that weekly video calls and shared spreadsheets are highly effective tools for maintaining participant engagement in long-term interventions. These adherence strategies not only ensure consistent participation but also enhance the reliability of the study’s outcomes, offering valuable insights for future research in olfactory rehabilitation.

## Figures and Tables

**Table 1:** Descriptive analysis per evaluation.

	Week 0	Week 12	Week 24	df	F	Test	*p*	SMD (1, 2)	SMD (1, 3)	SMD (2, 3)
***n***	11	11	11							
**Threshold (T), mean (SD)**	3.8 (3.3)	6.2 (3.8)	6.5 (3.4)	[2, 20]	3.368	ANOVA RM	0.055	-	-	-
**Discrimination (D), mean (SD)**	7.5 (3.4)	8.9 (3.2)	8.3 (3.1)	[2, 20]	0.647	ANOVA RM	0.534	-	-	-
**Identification (I), mean (SD)**	8.9 (1.3)	9.5 (2.1)	10.5 (2.3)	[2, 20]	2.190	ANOVA RM	0.138	-	-	-
**TDI, mean (SD)**	20.3 (5.6)	24.6 (4.3)	25.4 (6.2)	[2, 20]	5.115	ANOVA RM	0.016 *	0.869 (0.041 *)	0.859 (0.041 *)	0.136 (0.657)

* *p* < 0.05. *n*: sample size; df: degrees of freedom; F: F-statistic; Test: type of statistical test used; *p*: *p* value; SMD: standardized mean difference; TDI: composite score T + D + I.

**Table 2:** Descriptive analysis by sample.

	EG	CG	HG	df	F	Test	*p*	SMD (EG, CG)	SMD (EG, HG)	SMD (CG, HG)
***n***	11	11	11							
**Age**	34.55	33.00	32.55							
**Threshold (T), mean (SD)**	6.5 (3.4)	4.9 (3.5)	8.2 (2.5)	8	2.928	One-way ANOVA	0.069	−0.475 (0.457)	0.549 (0.457)	1.064 (0.055)
**Discrimination (D), mean (SD)**	8.3 (3.1)	10.5 (3.3)	11.5 (2.3)	8	3.391	One-way ANOVA	0.047 *	0.685 (0.205)	1.158 (0.042 *)	0.352 (0.706)
**Identification (I), mean (SD)**	10.5 (2.3)	10.6 (3.5)	13.5 (1.4)	8	4.903	One-way ANOVA	0.014 *	0.031 (0.996)	1.545 (0.026 *)	1.095 (0.032 *)
**TDI, mean (SD)**	25.4 (6.2)	26.0 (8.6)	33.2 (4.0)	8	4.819	One-way ANOVA	0.015 *	0.085 (0.972)	1.493 (0.024 *)	1.069 (0.040 *)

* *p* < 0.05. *n*: Sample size; df: degrees of freedom; F: F-statistic; Test: type of statistical test used; *p*: *p* value; SMD: standardized mean difference; TDI: composite score T + D + I.

## Data Availability

The datasets generated during and/or analyzed during the current study are available from the corresponding author on reasonable request.
